# Treatment of Nevus by a Solution of Iodine

**Published:** 1850-03

**Authors:** J. O. Bulteel

**Affiliations:** Plymouth


*Treatment of Nevus by a Solution of Iodine.* By Dr. J. O. Bulteel, Plymouth.

[Dr. Bulteel has used with the most satisfactory result a solution of iodine ℈j. in sp. vin. rect. ℥ss. He says:]

The preparation was applied freely once every day, not exciting the slightest constitutional derangement, and the disease every two or three days scaling itself, if I may use the expression, and thus disappearing *gradatim* till nothing more could be seen but two little spots of the size of a pin's head. The application at once arrests the growth of the nevus, and nothing but a regular daily application is needed for its final removal.—*Med. Gazette, Aug.* 24, 1849, *p.* 319.

